# Integrative Perspectives on Human Development: Dynamic and Semiotic

**DOI:** 10.1007/s12124-023-09783-y

**Published:** 2023-06-05

**Authors:** Tania Zittoun

**Affiliations:** Institute of psychology and education, University of Neuchâtel, FLSH - Espace Tilo Frey 1, Neuchâtel, CH – 2000 Switzerland

**Keywords:** Dynamic system, Semiotics, Integrative, Sociocultural, Lifecourse, Patterns

## Abstract

Werner Greve argues for an abstract and integrative theory of development; to progress toward such a theory, he suggests that evolutionary psychology can provide concepts for a processual approach to adaptation. To complement this perspective, I propose to start where the author finishes: the need to further qualify change, and to account for information. Considering ruptures and sense-making as cornerstones of a developmental approach, I recall that open dynamic approaches offer a meta-theoretical frame for an integrative developmental psychology, and that cultural approaches already and always account for meaning-making to start with. Assuming these two givens, a variety of integrative propositions account for stability and change; I present an historical example, the work of Gordon Allport, and a current one, our work as sociocultural psychologists, to show how the theoretical and heuristic interest of this proposition.

“It is worth looking for an abstract and integrative theory of development”, concludes convincingly Werner Greve in his paper (2023), after having identified the lack of progress in developmental psychology, and especially, the lack of integration of different perspectives. To progress toward such a theory, Greve highlights the need of a processual perspective to study development in the life span, and he finds in evolutionary approaches the abstract concepts that enable to account for self-regulation, continuity and change. From this perspective, the core process in development would be adaptivity - a concept that can encompass a diversity of mechanisms, at different levels of description, of how a person, an organism, a group or a function adjust to a problem, an issue, or a disturbance. This leaves of course open the work of clarifying the processes of adaptation in all its nuances; and also, the core problem of how to account for information in these processes.

## Integration in Developmental Psychology: Beyond and Below

The call for an integrative approach to human development in the lifespan is a very important one, and the direction proposed by Werner Greve seems at first sight conceptually and heuristically promising. In what follows, as sociocultural psychologist, I would however propose a complementary view on that issue. Rather than seeing the two problems identified by Greve – encompass the diversity of adaptations, and include information – as remaining, almost accessory problems, could they not be the starting point of our investigation? Should we not first consider that sudden changes and their consequences are primary – actually, this is the basis of life itself – and in addition, that one cannot produce a psychology without starting with the centrality of information, that is, meaning – meaning and sense making, their transmission and transformation?

Regarding the first problem, along his reflexion, Greve mentions processual approaches, and dynamic system theories seen mainly as mathematical approaches. But more fundamentally, I would say, processual and dynamic approaches impose a view by which even stability is a form of event – actually, an anomaly in the flow of time and matter – and these, fundamentally, invite us to observe how what flows can stabilise in dynamic wholes, which are always open to disequilibrium (Stenner, 2011; Valsiner et al., 2009; Witherington & Boom, 2019a). Werner Greve writes that insufficient attention had been given to open dynamic system approaches; I must however precise that many developmental psychologists historically decisive for the field did have a good knowledge of these approaches – Jean Piaget (Piaget, 1973; van Geert, 2000), obviously, but also, Gordon Allport (1960, 1963), a strong defender of open dynamic approaches against those based on close systems. Note, however, that they were building on the theoretical and conceptual strength of such approaches, not always on their mathematisation. Nowadays, most attempts to integrate and further contribute to developmental approaches, including cultural psychology, draw on open dynamic systems (Cole & Packer, 2016; Fogel et al., 2008; Neuman, 2003, 2014; Smith & Thelen, 2003; Valsiner et al., 2009; Witherington & Boom, 2019b). The advantage of such an approach is that it is meta-theoretical, and it admits both change and information, and so the limits of evolutionary theory – which cannot yet fully account for information – is not a problem within that frame; similarly, the fact that it requires a more precise declination of its core dynamics – say, equilibration – at various levels of analysis and different scales of changes is a given (Kelso, 1995, 2001).

The second problem, that of information, I address as sociocultural psychologist: in that approach, as in many other cultural, social and dialogical approaches, we consider that the world in which we live is made not only of things and organisms, it is made also and foremost, for humans, of meanings, that circulate through material arrangements, social discourses, movements, cultural artefacts, and in which people bathe from before they even are born. That is, any psychological developmental process is already and always semiotic, that is, cultural, in nature (Bruner, 2003; Neuman, 2014; Packer & Cole, 2020; Valsiner, 2000, 2021; Zavershneva & Van der Veer, 2018). And hence, a semiotic developmental psychology starts where Greve finishes: with dynamics of sense-making, from the early emergence of signs (Freud, 1950; Salvatore, 2016; Valsiner, 1997, 2001) to the building of churches, arts and political change (Gillespie, 2020; Marková, 2016; Valsiner, 2019, 2023; Wagoner et al., 2012; Zittoun, 2019). So, if meanings are part of the equation from the start, there is no remaining problem of information.

In other words, a sociocultural psychology complements the contributions of an evolutionary perspective both from above, and from below: from above, because it admits as a meta-theoretical frame open dynamic system approaches; and from below, because it does not have as unit of analysis people’s goals or activities, but the dynamic of sense-making. Of course, then, to remain a psychology, it has to have a solid conception of the developing person, situated in her world of things and others – and that is, a person feeling, dreaming, hoping and imagining (Perret-Clermont, 1992; Zittoun, 2022).

## On What Remains the Same and Yet Changes

There is however a problem I wish to explore more systematically; that of “adaptivity”. As Werner Greve writes, this notion has the power to account for stability through change, or *allostasi*s (Valsiner, 2000). Here I want to explore this stability through change at the level of how people address common daily issues, while respecting the centrality of change and the emergence of newness, and of sense-making, in two steps: I first propose a little return to the work of Allport, before second, presenting some of our current work.

Gordon Allport is usually remembered for his work on personality traits; but among his original contributions, one has to note his fundamental dynamic and developmental understanding of human beings (Allport, 1955, 1962, 1963). His understanding of motivation, or motives, responds to the call to know why people act at all, and what is meaningful to them, and to the core intuition that a psychology needs an energetic explanation that goes beyond the theory of drives. What characterises each person as unique, though, is the specific shape their action, goals and intention take – a motive is “any internal condition in the person that induces action or thought” (Allport, 1963, p. 196). In his open system view, Allport acknowledges that these motives can actually take various shapes, which characterise the person, as forms of patterns or motifs – yet these slowly evolve with time. And how is it that they evolve and apply to different life domains, to simple or more complex tasks, or back to simpler? Thanks to the principle of “functional autonomy”, which “*refers to any acquired system of motivation in which the tensions involved are not of the same kind as the antecedent tensions from which the acquired system developed*” (Allport, 1963, p. 196, italics original). Hence, Joe may become a politician because as child he admired his father and wanted to be like him (‘father identification’); eventually, the Oedipean motive gives room to another one, when as adult, politics may become part of his lifestyle, a passion in itself, etc. (Allport, 1963, pp. 228–229). Allport differentiates modalities of functional autonomy of motives: on the one side, “perseverative functional autonomy” designate routines, familiarities, automatism, which, we would say, demand little awareness of cultural mediation; on the other, “propriate functional autonomy” are more complex, or cultivated: “(1) ability often turns to interest; (2) acquired interests and values have a selective power; (3) self-image and life-style are organizing factors.” Without entering more in details here, this example is brought forward because Allport’s theoretical propositions correspond to the criteria discussed so far: these are based on an open-system dynamic approach, which enabled him to connect different levels of explanation, different scales of change, and some energetic principles, to account for the emergence of radical newness in a system; and these are admitting that people strive for meaning. Altogether, these constitute an original conception of adaptivity.

Second, we currently work with longitudinal data to account for the life of adults and older adults, both via diaries and repeated interviews. As any developmentalists, we wonder what develops and what remains the same, yet as sociocultural psychologist, we wonder how, in a given get of sociocultural, material cultural circumstances, people develop in their very unique ways. We draw on our past work identifying rupture and transitions as catalysed occasions of development and sense-making (Zittoun, 2006, 2012; Zittoun et al., 2003). We have used the term “dynamic pattern” to account for people’s unique style of acting, solving problems, imagining, or making sense (Cabra, 2021; Zittoun, 2019, 2022). These dynamic, psychological patterns precisely correspond to Allport’s earlier proposition: people’s way of acting or sense-making may change object, move across situations, become more concrete or more abstract, change modality of expression, become generalised… Here are two succinct examples from our current field works.

Ken, a diary writer during more than twenty years, was a young man during and after his studies in a film school, an intense film commentator and critic; he then found himself limited by years of unemployment – a long-standing rupture; he slowly gave up his dream to work as film director, and in his diary, he progressively stopped writing film critics, to mention more his activity as a fantasy-fair fan. However, he also started to write more about national and international affairs, using his analytical sense in that domain, thus becoming a regular acerb critic of politics. These critics can be seen, in some way, a dynamic pattern that move across domains and became more general, and perhaps enabled to maintain himself in terms of duress and to make sense of his situation^1^ (Zittoun et al., Submitted).

Agathe is a 86 years old woman that we repeatedly interviewed^2^, and who explained that, as they were seven children when she grew up, they had to learn to “stand for themselves” – and so, now, she stands for her rights in any circumstances: writing to a prime minister if she feels she has been unjustly limited of her pension after her husband left her, or to the head of the Swiss national railway company, after calculating that she has been unfairly reimbursed her train abonnement during the covid pandemic. She clarifies always that she does not need the money, but that it is a matter of principle. Her deep sense of justice, together with her sense of legitimacy, appears to be a dynamic pattern in her life, emerging in her childhood, cultivated, and on which she draws on at every blow – rupture – of life.

Of course, the examples are very superficially presented; yet what they mean to convey, is that a sociocultural developmental view, supported by an open dynamic approach enables us, via concepts such as rupture, transition and dynamic patterns, to account for variations in adaptivity and sense-making, and for the emergence of newness in people’s personal engagements.

## Openings

Greve (2023) proposes a diagnostic of the current fragmentation of developmental psychology, and a cure, to recur to evolutionary psychology. With many others, I tend to concur with the diagnostic; but I believe that the response overlooks alternative attempts to overcome the crises. In particular, I proposed to consider instead approaches that take seriously two aspects that are undermined by such a proposition: the centrality of change and disruption, and the primacy of sense-making, which can be considered as cornerstones of any attempt to account for the development of human beings in our complex societies. Many approaches, historically, have admitted these principles (Elder, 1996; Valsiner, 2005; Valsiner & Van der Veer, 2000) – here I mainly mentioned Gordon Allport, and it is also the case in current sociocultural psychology (Cole & Packer, 2016; Kirschner & Martin, 2010; Neuman, 2014; Salvatore, 2016; Valsiner, 2021; Wagoner et al., 2021). In any case, approaches that admit an open-dynamic system approach and the centrality of meaning, notably - but not only - through a semiotic perspective, present many advantages.

First, they propose parsimonious explanations for the intra- and interlevel dynamics of change in a complex system, for instance by articulating sociogenesis with microgenesis, or by inviting to consider the interrelation between social discourses and personal sense-making (Cole & Packer, 2016; Duveen & Lloyd, 1990; Kelso, 2001; Marková, 2016; Psaltis et al., 2015). Second, they are empirically generous: because of their need to account for complex phenomena, they are methodologically ecumenic, admitting a large range of quantitative and qualitative data and their articulation, including diaries, self-writing, repeated observations, ethnographies, etc. (Allport, 1942; Brinkmann, 2012; Gillespie et al., Submitted; Valsiner et al., 2009; Zittoun & Gillespie, 2022). Third, they provide with a set of meta-concepts that enable to overcome the fragmented nature of the field, and put to the fore the similarities, differences and complementarities between concepts (e.g., typically, around the notion of crises, conflict, bifurcation, rupture, etc.). And fourth, they invite us to bring together, and again, compare and possibly integrate, concepts that enable us to capture the complexity of stability and change around specific issues in human conduct (e.g., schemes, motives, or patterns) (Zittoun & Cabra, 2022).

Developmental psychology may be a complex and heterogeneous field, with a centrifugal tendency to fragment as it expands; but to create a centripetal movement, one may not have to turn to evolutionary models; the history of psychology is rich, and its theoretical apparatus powerful enough, to find other ways to work towards more integration – theoretical work and dialogue, as practiced in this integrative journal, being good starting practices.

## Footnotes

1 The data was primarily collected by Marco Bernal and collaboratively analysed with him and Alex Gillespie.

2 The interviews were made by Aurora Ruggeri and Isabelle Schopefer.
